# Daptomycin as supportive treatment option in patients developing mediastinitis after open cardiac surgery

**DOI:** 10.1186/1749-8090-7-81

**Published:** 2012-09-04

**Authors:** Florian Weis, Jens Heyn, Christian L Hinske, Ferdinand Vogt, Marion Weis, Felix Kur, Christian Hagl, Andres Beiras-Fernandez

**Affiliations:** 1 Department of Anaesthesiology, University of Munich (LMU), Marchioninistrasse 15, 81377, Munich, Germany; 2 Department of Cardiac Surgery, Grosshadern University Hospital, LMU-University, Marchioninistrasse 15, D-81377, Munich, Germany; 3 Department of Thoracic and Cardiovascular Surgery, University Hospital, JW Goethe-University, Theodor-Stern-Kai 7, 61590, Frankfurt, Germany

**Keywords:** Mediastinitis, Cardiac surgery, Daptomycin

## Abstract

**Background:**

Mediastinitis is a severe complication after cardiac surgery. While improvement of prophylaxis and of medical and surgical therapy has reduced its incidence, the treatment of mediastinitis continues to be a challenging problem. Within this study, we report the successful use of daptomycin as supportive therapy in patients developing mediastinitis after open cardiac surgery.

**Methods:**

The records of 21 consecutive patients who developed mediastinitis after cardiac surgery were retrospectively reviewed. After diagnosis, all patients received surgical debridement and antibiotic therapy with daptomycin. All patients were followed up to death or discharge.

**Results:**

Clinical improvement after combined surgical and antibiotic therapy with daptomycin was found in 90.5% of the patients. The median time until clinical improvement occurred was 5 [4/6] days. Daptomycin was well-tolerated and no major adverse events during therapy were observed observed.

**Conclusions:**

This study provides new and helpful information regarding the beneficial use of daptomycin as supportive treatment option in patients developing mediastinitis after cardiac surgery.

## Background

Sternal wound infection after cardiac surgery is a serious and potentially life-threatening complication. The exact pathologic mechanism underlying this infection remains unclear; patients’ pre-operative co-morbidities, as obesity, diabetes, peripheral arterial disease or chronic obstructive pulmonary disease may play an important role [1,2].

In up to 5% of patients undergoing sternotomy, mediastinitis occurs after surgery [3]. Mediastinitis is associated with high in-hospital [4] and long-term mortality rates and significant additional costs [5]. The treatment of mediastinitis usually consists of combined surgical and antibiotic [6]. So far, newly developed antibiotics have not been evaluated regarding their effects as supportive treatment option to surgical debridement [7].

Daptomycin (Cubicin^©^, Novartis Pharma AG, Nuremberg, Germany), a cyclic lipopeptide antibiotic, is a newly and useful drug against infections with multidrug-resistant gram-positive organisms. Daptomycin has been successfully used in the treatment of endocarditis and complicated cardiac device infections [8]. We report the beneficial use of daptomycin as supportive traetment option to surgical debridement in 21 patients who developed mediastinitis after cardiac surgery.

## Methods

We retrospectively reviewed the records of 21 consecutive patients who developed mediastinitis after cardiac surgery followed by combined surgical debridement and antibiotic therapy with daptomycin. During initial operation, the included patients received 3 x 1.5 g Cefuroxime as antibiotic prophylaxis.

The diagnosis of mediastinitis was defined by means of clinical assessment, CT-scans, laboratory values, and microbiologic analysis. In the circumstances of mediastinitis, the patients usually developed fever, tachycardia, and leucocytosis. In addition to the responsible microorganisms, we analysed laboratory findings, risk factors, drug compatibility, and the outcome.

All patients underwent aggressive surgical exploration followed by the closed irrigation technique (CIT) and received daptomycin intravenously (initial dosage of 6 mg/kg on the first day and 4 mg/kg, the following days). CIT consists of wound debridement, sternal stabilization and closed irrigation of the mediastinum and pericardium with saline or antiseptic solutions. Sternal refixation occurred mostly using a Robicsek technique as described before [9]. Tissue cultures and cutaneous swabs were obtained in all patients during operation.

The patients were followed up to either death or discharge. Therapy was considered unsuccessful if recurrence of infection occurred, indicated by persistent purulent secretion and wound dehiscence.

Statistical descriptive analyses of the results were expressed as median and 25th to 75th percentile or as numbers with related percentage. All analyses were performed using SPSS software (version 15.1 for Windows, SPSS, Inc, Chicago, Ill, USA).

## Results

21 patients developed mediastinitis after cardiac surgery. These patients had a median age of 66 [56.5/76] years. 20 of the patients (95.2%) were male and 1 patient (4.8%) was female. Risk factors for wound complications were similar to those reported by others [10]. Six patients (28.6%) were diabetic and required either insulin or oral agents, seven patients had chronic renal failure (33.3%), and two patients (9.5%) showed COPD pre-operatively (Table 1). The median BMI of these patients was 24.7 [23.2/27.8]. 10 of the included patients received corticosteroids (8 patients received hydrocortisone and 2 patients prednisolone due to organ transplantation).

**Table 1:** Preoperative risk factors for mediastinitis after cardiac surgery

**Risk factors**	**Number (%)**
Mitral regurgitation	13 (61.9)
Peripheral vascular disease	12 (57.1)
Aortic stenosis	7 (33.3)
Dialysis dependent renal insufficiency	7 (33.3)
Diabetes	6 (28.6)
Internal mammary artery bypass	3 (14.3)
Chronic obstructive pulmonary disease	2 (9.5)

Procedures performed included aortic valve replacement (5 patients), coronary artery bypass (4 patients), heart transplantation (3 patients), left ventricular assist device (3 patients), biventricular assist device (2 patients), mitral valve repair (2 patients), and implantation of a defibrillator (2 patients). The Berlin Heart Excor® (Berlin Heart, Berlin, Germany) was used as ventricular assist device. (Table 2).

**Table 2:** Primary heart surgery prior to mediastinal infection

**Primary heart surgery**	**Number (%)**
Aortic valve replacement	5 (23.8)
Coronary artery bypass	4 (19.0)
Heart transplantation	3 (14.3)
Left ventricular assist device	3 (14.3)
Biventricular assist device	2 (9.5)
Mitral valve repair	2 (9.5)
Implantation of a defibrillator	2 (9.5)

Diagnosis was established by smear, blood cultures, CT-scan and clinical symptoms. Microbiological evaluation of these samples revealed Staphylococcus aureus (8 patients), various gram-positive organisms (5 patients), Enterococcus faecium (3 patient), and methicillin-resistant Staphylococcus aureus (MRSA – 3 patients). A pathogen could not be identified in 2 patients, due to the fact that antibiotic therapy was initiated after the appearance of first symptoms and before the diagnosis of mediastinitis was established (Table 3). These two patients died during the hospital stay because of sepsis and multi-organ failure related to severe infection. In 14 cases (66.7%) the pathogen was only sensible to vancomycin, linezolid or daptomycin.

**Table 3:** Organisms causing the mediastinitis

**Organism associated with mediastinitis**	**Number (%)**
Staphylococcus aureus	8 (38.1)
Gram-positive organisms	5 (23.8)
Enterococcus faecium	3 (14.3)
MRSA	3 (14.3)
No germ isolated	2 (9.5)

All patients underwent surgical debridement combined with drainage in 5 patients and removal of the implanted device in 4 patients. The removed devices were implantable cardioverter defibrillators; ventricular assist devices had not to be removed. Microbiological analysis of the removed materiel did not show any bacteriological population.

Surgical debridement was combined with calculated antibiotic therapy. Therapy was initiated with ureidopenicillins (in 9 cases as monotherapy and in 6 cases as combined therapy), carbapenems (in 7 cases as monotherapy and in 12 cases as combined therapy), cephalosporins (in 3 cases as combined therapy), or others (in 11 cases as combined therapy). Antibiotic therapy was changed to Daptomycin in all patients after 2 [1/3] days according to the antibiogram or in cases of disease progression.

**Figure 1:**
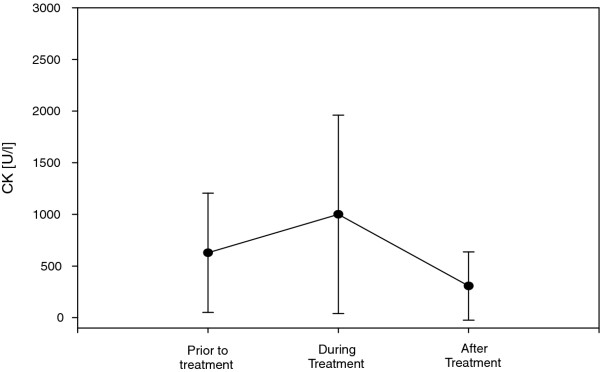
Creatine Kinase (CK) values before, during and after therapy with daptomycin.

**Figure 2:**
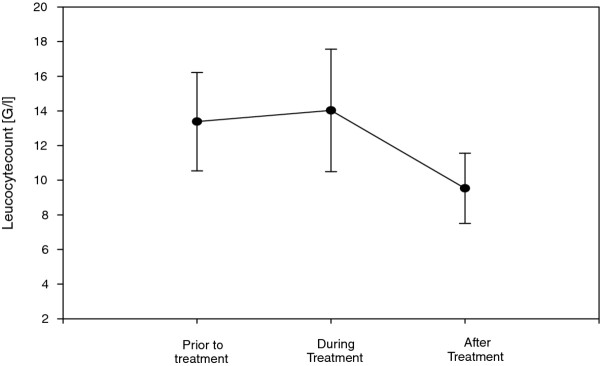
Leucocyte values before, during and after therapy with daptomycin.

Clinical improvement after therapy with daptomycin was detected in 90.5%; in 17 patients (81%) eradication without residues was achieved, in two patients (9.5%) eradication with residues was detected, and in two patients the therapy was unsuccessful (Table 4). The median until clinical improvement occurred was 5 [4/6] days. During therapy, daptomycin lead to mild increase in creatinine kinase, and values of leukocytes. These increased values declined distinctly after therapy (Figure 1 and Figure 2). No major adverse events during antibiotic therapy with daptomycin were detected; only a mild allergic reaction occurred in a single case. Median duration of daptomycin therapy was 8.5 [7/15] days.

**Table 4:** Clinical alteration after therapy with daptomycin

**Therapeutical success**	**Number (%)**
Healing without residues	17 (81.0)
Healing with residues	2 (9.5)
Unsuccessful	2 (9.5)

## Discussion

In spite of improvements in surgical techniques and medical therapy, the frequency of mediastinitis has remained stable during the last years [11]. Therefore, therapy of mediastinitis after cardiothoracic surgery seems to be a challenging problem [3].

Staphylococcus aureus is the most common isolated germ in patients developing mediastinitis [12]. Unfortunately, Staphylococcus aureus has become an evolving management problem, because of an increasing number of antibiotic resistance and MRSA infections which causes high rates of morbidity and mortality [12,13].

Vancomycin is still the antibiotic therapy of choice for the treatment of infections caused by MRSA. However, the therapy with vancomycin may be contraindicated in a number of cases (e.g. renale failure) and ineffective in numerous cases because of limited tissue penetration [14]. Furthermore, Staphylococcus aureus (including MRSA) exhibits a declining susceptibility to vancomycin during the last years [15]. Thus, the need for alternative antibiotic therapies as supportive therapy after surgical debridement has become apparent [15].

An alternative antibiotic option seems to be linezolid, because linezolid eradicates MRSA better than vancomycin in complicated skin or soft-tissue infections caused by MRSA. Furthermore, linezolid reduces the length of hospitalization in these patients [16]. Despite the advantages of linezolid, concerns about safety and costs often limit its use.

Daptomycin is a cyclic lipopeptide antibiotic with bactericidal activity against gram-positive organisms in a concentration-dependent manner [8]. In patients with (gram-positive) wound infection, daptomycin is useful in more than 90% [17] which is comparable to success rates found for vancomycin (87%) and linezolid (93%). Furthermore, eradication of bacterial infections by daptomycin amounts 96% in the case of MRSA, 92% in the case of coagulase-negative staphylococci, and 88% in the case of Enterococcus species [16,17].

The efficiency of daptomycin is consistent with the pharmacokinetic profile of this drug. Daptomycin exhibits excellent penetration of subcutaneous tissue levels in healthy volunteers (averaging 74% of plasma levels) and inflammatory tissues (averaging nearly 70% of plasma levels) [17]. Although daptomycin penetrates the tissue rapidly, disappearance is relatively slow, with an elimination half-life of more than 17 hours [6,16,17].

Daptomycin is highly effective in the treatment of bacteraemia and endocarditis caused by MRSA and several reports document its effectiveness in infections related to cardiac surgery [8,18]. Based on these observations, we analysed the role of daptomycin as new treatment option as supportive treatment to surgical therapy in patients developing mediastinitis after cardiac surgery. Within our study, we found clinical success in more than 90% when daptomycin was combined with surgical therapy. The patients responded to daptomycin therapy commonly after 5 days. Daptomycin was well tolerated in the present study which is in accordance to previous studies. The patients may benefit by the fact, that daptomycin is able to penetrate into device-adhering biofilms better than other antibiotics [6]. However, controlled prospective studies are necessary to evaluate the optimal surgical and antibiotic regime in these patients

## Conclusion

This study provides new and helpful information regarding the beneficial use of daptomycin as supportive treatment option in patients developing mediastinitis after cardiac surgery.

## Competing interests

ABF is Advisor of Fresenius Biotech. ABF has received fees from Merck, Astellas and Orion Pharma for being an advisory board member. ABF has received travel grants and/or honoraria from Sanofi, Fresenius Biotech, Novartis, BG Medicine and Orion Pharma for lectures.

## Authors’ contributions

FW, JH, CLH, and AB designed the study and drafted the manuscript. CH and MW participated in the design of the study and revised the manuscript critically. FW, JH, CLH, FV, and FK collected the data and performed the statistical analysis. All authors interpreted the data, read the final manuscript and approved it.
